# Diacylglycerol Kinases and Its Role in Lipid Metabolism and Related Diseases

**DOI:** 10.3390/ijms252313207

**Published:** 2024-12-09

**Authors:** Yishi Liu, Zehui Yang, Xiaoman Zhou, Zijie Li, Nakanishi Hideki

**Affiliations:** Key Laboratory of Carbohydrate Chemistry and Biotechnology, Ministry of Education, School of Biotechnology, Jiangnan University, Wuxi 214122, China; yzh3210@163.com (Z.Y.); xiaoman@jiangnan.edu.cn (X.Z.); lizijie@jiangnan.edu.cn (Z.L.); hidekinakanishi@hotmail.com (N.H.)

**Keywords:** diacylglycerol kinase, lipid, phosphorylate diacylglycerol, phosphatidic acid

## Abstract

Lipids are essential components of eukaryotic membranes, playing crucial roles in membrane structure, energy storage, and signaling. They are predominantly synthesized in the endoplasmic reticulum (ER) and subsequently transported to other organelles. Diacylglycerol kinases (DGKs) are a conserved enzyme family that phosphorylate diacylglycerol (DAG) to produce phosphatidic acid (PA), both of which are key intermediates in lipid metabolism and second messengers involved in numerous cellular processes. Dysregulation of DGK activity is associated with several diseases, including cancer and metabolic disorders. In this review, we provide a comprehensive overview of DGK types, functions, cellular localization, and their potential as therapeutic targets. We also discuss DGKs’ roles in lipid metabolism and their physiological functions and related diseases.

## 1. Introduction

**Figure 1:**
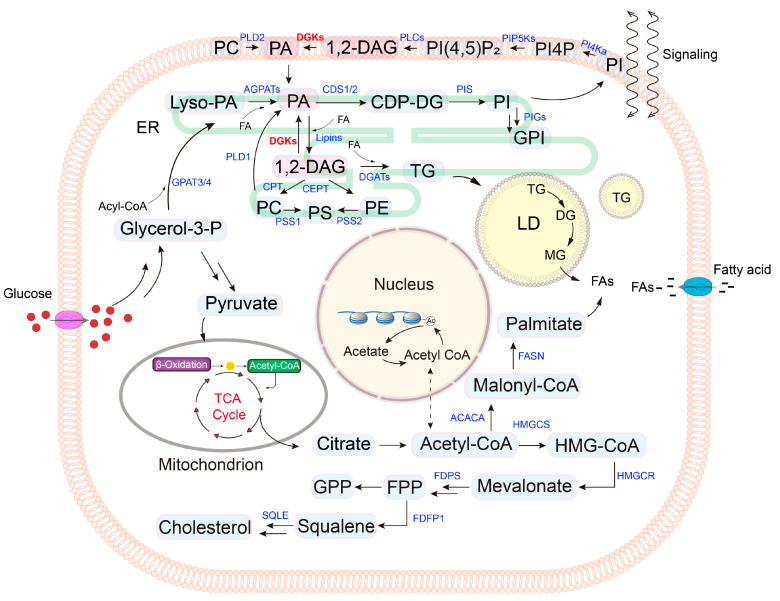
Overview of the enzymatic pathway of lipid metabolism in major organelles of mammalian cells. The enzymes are labeled blue except DGKs (Red). The lipids or their intermediates are black with light blue filling. PA and 1,2-DAG are black with pink filling, which represents a major hub for several metabolic pathways.

Glycerophospholipids (GPL), sphingolipids, and sterols are the three major classes of lipids in eukaryotic membranes. These lipids are not only fundamental components of cell membranes but also serve as essential carriers of energy and signaling molecules. Structural lipids are primarily synthesized in the endoplasmic reticulum (ER), from which they are subsequently transported to the membranes of other organelles [1]. Although the biosynthetic pathways of phospholipids have been well established over several decades, these intricate metabolic processes remain complex [1]. In the first step of de novo lipid biosynthesis, the glycerol-3-phosphate acyl transferase family transfers fatty acyl chains to glycerol-3-phosphate (G3P) to form lysophospholipids [2,3,4]. Subsequently, acylglycero-phosphate acyltransferases catalyze the acylation of lysophospholipids to produce phosphatidic acid (PA) [5,6], an important intermediate step in lipid metabolism. PA can then be utilized for the synthesis of other phospholipids or converted into various other lipids, such as phosphatidylinositol (PI), phosphatidylcholine (PC), phosphatidylethanolamine (PE), phosphatidylserine (PS), triacylglycerol (TG) and lipid droplets (Figure 1) [7,8,9,10].

Lipid signaling is also essential in cellular communication, coordinating numerous physiological processes through complex signaling networks [11,12,13,14,15]. In the phosphatidyl inositol (PtdIns) cycle, the addition or removal of phosphate groups on lipids is essential for cellular signal transduction [16]. Diacylglycerol kinases (DGKs) form a conserved enzyme family that phosphorylates diacylglycerol (DAG) to form phosphatidic acid (PA) [17,18,19]. Both DAG and PA serve as essential lipid second messengers, modulating various cellular processes, including signal transduction and membrane dynamics [20,21,22]. Additionally, these lipids facilitate the activation of several proteins, such as protein kinase C (PKC), RAS activator proteins, mammalian target of rapamycin (mTOR), and tyrosine kinases [23,24,25,26].

**Figure 2:**
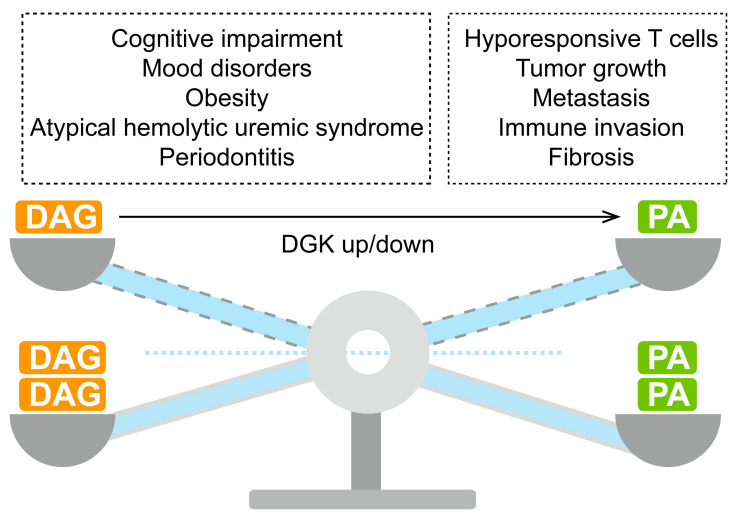
Human diseases related to DGK activity. DGKs regulate the conversion of diacylglycerol (DAG) to phosphatidic acid (PA), both of which play critical roles in various cellular processes. Disruptions in DAG/PA homeostasis resulting from DGK dysfunction are associated with a range of diseases.

PA can be converted into DAG through lipin-catalyzed reactions, and DAG can be further utilized to synthesize triacylglycerol (TG), which is stored in lipid droplets. This process is reversible, and regulation of DGK affects the balance between PA and DGA, potentially contributing to disease development (Figure 2). Lipid droplets contribute significantly to cellular homeostasis by alleviating cellular stress, regulating energy balance, providing membrane lipid precursors, supporting lipid trafficking, and serving as docking sites for protein storage and degradation [27,28]. Aberrant lipid droplet accumulation and mutations in lipid-droplet associated genes are linked to various diseases, including obesity, lipodystrophy, non-alcoholic fatty liver disease, cancer, and cardiovascular disease [27]. Additionally, PA is catalyzed by CDS and PIS proteins to produce PI, a precursor for synthesizing GPI-anchored proteins. Deficiency in GPI leads to inherited GPI deficiency (IGD), associated with conditions like paroxysmal nocturnal hemoglobinuria, multiple congenital anomalies-hypotonia-seizures syndrome, developmental delay/intellectual disability, inflammation, hypotonia, and hyperphosphatasia with mental retardation syndrome/Mabry syndrome [29]. Moreover, DAG functions as a crucial second messenger that regulates multiple signaling pathways, such as cell proliferation, survival, and migration, primarily through its interaction with protein kinase C (PKC) [30,31]. The DAG/PKC pathway has also been linked to tumorigenesis [32].

The study of DGKs in these processes holds significant implications for understanding cellular physiology and the pathogenesis of various diseases. This review focuses on the multifaceted roles of DGKs, including their types, cellular localization, involvement in lipid metabolism, physiological functions, and potential as therapeutic targets.

## 2. Types of Diacylglycerol Kinase and Their Structural Features

**Figure 3:**
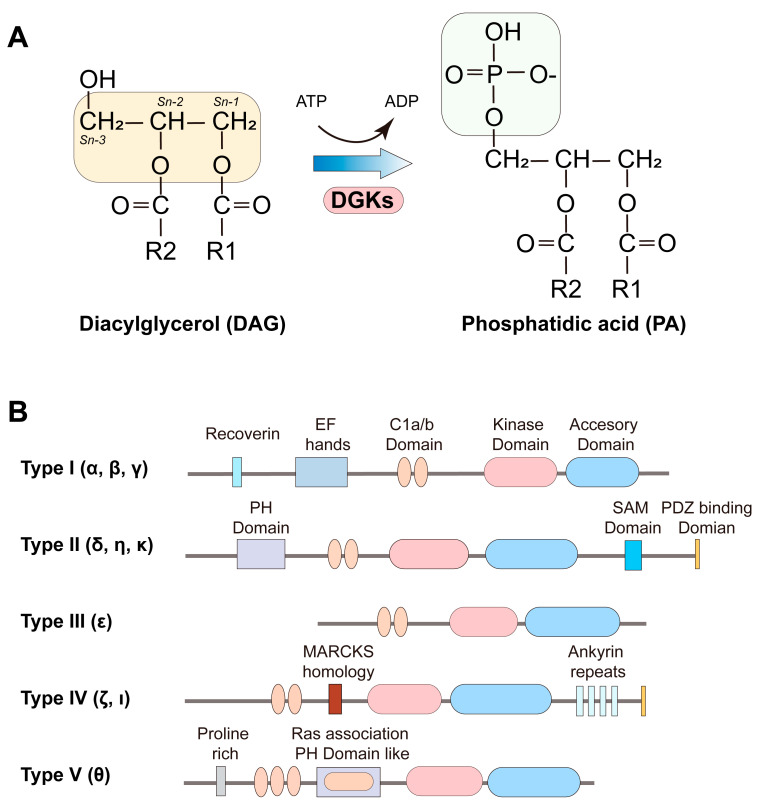
Structures and classification of mammalian DGKs. (**A**) DGK phosphorylates diacylglycerol (DAG) into phosphatidic acid (PA). (**B**) DGK are grouped into five types, based on the presence of conserved domains.

In mammals, ten DGK isoforms are classified into five subtypes based on functional domains (Figure 3) [33,34,35]. All DGKs possess a catalytic domain and at least two C1 domains (cysteine-rich domains). The catalytic domain contains two parts, a catalytic subdomain and an accessory subdomain. A highly conserved Gly-Gly-Asp-Gly motif in the catalytic domain that acts as the ATP binding site is essential for enzymatic activity [22,36]. Type I DGKs (α, β, γ) contain an N-terminal calcium-sensitive recoverin homology (RVH) domain and two EF-hand motifs [22,37,38,39]. The RVH domain of DGKα resembles the N-terminus of the recoverin calcium receptors and is crucial for calcium-dependent activation [40]. Removing the RVH domain impairs this activation, and deleting both the RVH and EF hands leads to constitutive activation of DGKα, indicating that the RVH domain senses calcium, while the EF hand suppresses kinase activity [41]. These domains may collaborate to regulate DGKα function through interactions with its C1 and catalytic domains. Additionally, calcium binding to the EF hands can facilitate the translocation of DGKα to the plasma membrane [42]. While the functions of the RVH and EF hands in DGKβ and DGKγ remain unclear, these isoforms’ EF hands have lower calcium affinity, suggesting minimal calcium regulation [43]. Interestingly, deleting both domains in DGKγ triggers translocation and cytoplasmic protrusions in a neuroblastoma cell line, hinting that calcium regulation may also apply to other Type I DGKs [44].

Type II DGKs (δ, η, κ) contain pleckstrin homology (PH) domains, a large family of lipid-binding domains, which interact with lipids or proteins [22,35,45,46]. Additionally, DGKδ and DGKη possess a sterile α motif (SAM) at their C-termini, likely aiding oligomerization through zinc binding [47,48]. DGKκ lacks a SAM domain but includes a C-terminal motif that may interact with type I PDZ domains [49].

Type III DGK (ε) is the smallest member of the DGK family, with a molecular weight of 64 kDa, and is unique in lacking additional regulatory domains [50]. DGKε is vital in the PI cycle, catalyzing the conversion of DAG to PA with a preference for sn-2-arachidonoyl DAG species containing 18:0/20:4 acyl chains [50,51]. This substrate specificity implies a role for DGKε in polyphosphoinositide (PPIns) metabolism, where arachidonoyl-DG is efficiently incorporated [52,53]. Knockout studies of DGKε have demonstrated a reduction in PI/PIPn enriched with these acyl chains [54]. Furthermore, DGKε is linked to the production of 2-arachidonoyl glycerol (2-AG), an endocannabinoid involved in brain signaling [51,55]. The N-terminal segment of human DGKε plays a key role in regulating the phosphatidylinositol cycle, influencing its rate without altering the acyl chain composition of its lipid intermediates [53]. S-palmitoylation at the N-terminal transmembrane domain of DGKε affects its localization primarily to the endoplasmic reticulum and Golgi apparatus and modulates its activity [56].

Type IV DGKs (ζ, ι) feature a MARCKS (myristoylated alanine-rich protein kinase C substrate) homology domain, ankyrin repeats, and a PDZ-binding motif [22,35]. The MARCKS domain in DGKζ includes four serine residues that may be phosphorylated by protein kinase C (PKC) α [57]. In certain cells, phosphorylation of these residues causes DGKζ to translocate to the nucleus, while in Jurkat T cells, mutating these serines to alanines prevents DGKζ translocation to the plasma membrane [57,58]. The PDZ-binding and ankyrin domains facilitate protein-protein interactions. The PDZ-binding domain controls DGKζ’s interaction with Sorting Nexin-27 (SNX27), which is necessary for vesicle trafficking, and with γ1-Syntrophin, which influences DGKζ’s subcellular localization in neurons and muscle cells [59,60,61,62]. The ankyrin domain regulates DGKζ’s interaction with the leptin receptor in the hypothalamus, possibly affecting leptin signaling [63]. The roles of the MARCKS and ankyrin domains in DGKι are unclear, but its PDZ-binding domain enables interaction with PSD-95, a neuronal scaffolding protein [64].

Type V DGKs (θ) contain an N-terminal proline-rich domain, a PH domain, and a Ras-association domain within the PH domain [22,35]. Mutations within the PH and proline-rich domains of DGKθ reduce its enzymatic activity [65]. Additionally, N-terminal phosphorylation of DGKθ promotes membrane binding and enzymatic activity [66].

## 3. Expression and Subcellular Localization of DGKs

The human DGK family exhibits diverse properties in enzymatic activity, tissue distribution, cellular expression, and binding partners, which are essential for cell growth and development, immune responses [21], glucose metabolism [67], and cancer progression [68,69,70,71]. For instance, many DGKs are highly expressed in the brain and the immune system, indicating specialized roles within these tissues [72,73,74]. Studies have shown that DGKα and DGKζ negatively regulate T-cell receptor (TCR) response [75,76,77]. Recent findings reveal that DGKα, DGKδ, DGKε, and DGKζ are ubiquitously expressed, whereas DGKθ is present at low levels, and DGKβ and DGKι are absent in human immune cells [78]. DGK localization is governed by distinct regulatory domains and protein interactions, enabling the modulation of various cellular processes in specific subcellular regions [20,79,80]. The expression and localization of certain DGK isoforms are dynamically regulated in response to external signals and varying metabolic demands. The main subcellular localization of human DGK isoforms is presented in Table 1.

## 4. DGKs in Lipid Metabolism and Signal Transduction

Lipids are not only essential components of cell and organelle membranes but also serve as critical energy storage molecules for cells [81,82,83]. DAG includes over 50 molecular species with diverse acyl chain combinations at sn-1,2, sn-1,3, or sn-2,3 positions (Figure 3A) [83,84,85]. DAG acts as a key intermediary between lipid metabolism and intracellular signaling. Since DAG is primarily metabolized by DGKs to produce PA, DGKs play a critical role in balancing DAG and PA, thereby influencing lipid metabolism [17]. In the PI signaling pathway, both DAG and PA function as vital secondary messengers. Unlike PA, DAG can freely translocate between membrane leaflets without requiring a flippase enzyme [86]. DAG can be generated through hydrolysis of the phospholipid headgroup or by the dephosphorylation of PA via phosphatic acid phosphatases (LIPINs) [84,87,88]. On the cell membrane, the DGK substrate is DAG-containing acyl chains at sn-1 and sn-2 positions (1,2-DAG), derived from phosphatidylinositol 4,5-bisphosphate (PIP2) and has been identified as an intracellular signaling molecule that activates several proteins, including PKC, Unc-13, RasGRP, and transient receptor potential channels [37,89]. Notably, DGKε specifically phosphorylates DAG with an arachidonoyl group (C20:4), the main product of phosphatidylinositol phospholipase C-mediated phosphoinositide hydrolysis from the PI pathway [90]. During glycosylphosphatidylinositol (GPI) anchor biosynthesis, the PI moiety is typically 1-stearoyl, 2-arachidonoyl PI(C18:0/C20:4) [91], suggesting possible connections between DGKε and GPI anchor biosynthesis.

In energy metabolism, 1,2-DAG acts as an intermediate in triglyceride (TG) synthesis [92]. De novo PA synthesis occurs through the G3P and lysophosphatidic acid pathway, the primary route catalyzed by lysophosphatidic acid acyltransferase (LPAAT) and phospholipase D (PLD)-mediated hydrolysis of phospholipids [84]. DAG can be a precursor of TAG synthesis by DAG acyltransferase (DGAT) and a product of TG hydrolysis by adipose triglyceride lipase (ATGL) [93,94]. DGKs phosphorylate 1,2-DAG to produce PA, a reaction reversible by PA phosphatase, which dephosphorylates PA to generate DAG [17,95]. PA is subsequently converted into various lipid metabolic intermediates or signaling molecules.

## 5. Physiological Functions of DGKs

DGKs are implicated in numerous signaling pathways, such as those involved in development, neural and immune responses, cytoskeleton reorganization, glucose metabolism, and carcinogenesis [71,75]. The primary DGK-related diseases and symptoms are summarized in Table 2. DGKα, along with DGKζ, is recognized for its role in promoting T-cell anergy, a process that limits T-cell activation in the tumor microenvironment and supports tumor survival via PA generation [96,97,98]. This dual functionality poses challenges for cancer therapy but also underscores the potential of targeting DGKα to improve treatment outcomes, particularly in CAR-T cell therapies [99]. DGKα plays an essential role in the programmed cell death-1/ligand-1 (PD-1/PD-L1) axis, significantly limiting the Ras/extracellular signal-regulated kinase (ERK) pathway, which is crucial for activator protein-1 (AP-1) activation [100,101,102]. Pharmacological inhibition of DGKα has been shown to synergize with PD-1 targeted therapies to restore T cell activation [100]. Recent findings suggest a macrophage-specific immune-regulatory role for DGKα. In bone marrow-derived macrophages (BMDMs) from wild-type and knockout (KO) mice, KO macrophages showed increased responsiveness to various stimuli, including LPS, IL-4, and MCP-1. In vivo, Dgka^−/−^ mice exhibited reduced wound sizes in full-thickness burn models [103].

DGKβ is primarily expressed in the brain, particularly in regions such as the striatum and hippocampus [128,129,130], with peak expression around 14 days post-birth, coinciding with synaptic maturation [129]. Notably, knockout studies show DGKβ is essential for emotional responses and long-term memory, as its absence results in mania-like behaviors [131,132]. DGKβ induce filopodium-like protrusions in COS-7 cells, with this effect linked to its plasma membrane localization and colocalization with F-actin [133]. Additionally, DGKβ also promotes neurite outgrowth and spinogenesis in neurons by activating mTORC1 through a kinase-dependent mechanism [134].

DGKγ is predominantly expressed in cerebellar Purkinje cells and plays a critical role in neuronal function and cerebellar signaling pathways [135,136]. DGKγ is crucial for cerebellar long-term depression (LTD) and dendritic development through protein kinase C γ (PKCγ) regulation, as DGKG-KO mice exhibit impaired motor coordination, reduced LTD, and significant dendritic retraction of Purkinje cells. Treatment with the cPKC inhibitor Gö6976 reversed this dendritic retraction [137]. Recently, DGKγ has also been implicated in tumor angiogenesis and the differentiation of immunosuppressive regulatory T cells in hepatocellular carcinoma (HCC). Under hypoxic conditions, HIF-1α directly activates DGKG transcription, elevating DGKG levels, which subsequently recruit ubiquitin-specific peptidase 16 for ZEB2 deubiquitination. This process enhances TGF-β1 secretion, facilitating tumor angiogenesis and regulatory T-cell differentiation [138].

DGKδ is the primary isoform in skeletal muscle and adipose tissue, where reduced expression is associated with insulin resistance in diabetic individuals [139,140,141]. DGKδ deficiency exacerbates hyperglycemia-induced peripheral insulin resistance and plays a critical role in the progression of type 2 diabetes [140]. In contrast, DGKδ overexpression in cultured myotubes enhances both basal and insulin-stimulated glucose uptake [141]. Furthermore, DGKδ interacts with serotonin transporter (SERT), inducing its degradation through the Praja-1 ubiquitin ligase-proteasome system in an activity-dependent manner [142]. Brain-specific DGKδ knockout mice exhibited obsessive-compulsive disorder-like behaviors sensitive to SERT inhibitor, and SERT protein levels are markedly elevated in the DGKδ-deficient brain [142].

The DGKη gene has been associated with bipolar disorder (BPD) and is located in the BPD linkage region on 13q14 [110,143,144,145]. DGKη-knockout mice exhibit lithium-sensitive BPD-like behaviors and increased dopaminergic activity, underscoring its relevance in BPD [146,147]. Additionally, DGKη interacts with C-Raf and B-Raf (mitogen-activated kinase (MAPK) kinase (MAPKKK)) in response to epidermal growth factor (EGF), regulating the Raf–MAPK pathway [148]. Under stress conditions, DGKη translocates to specific membrane structures and associates with apoptosis signal-regulating kinase 3 (ASK3) in an osmotic shock-dependent manner, suggesting a specialized stress response role [149]. DGKη knockout significantly increases dopamine (DA) levels in the midbrain and cerebral cortex, with elevated phosphorylated dopamine transporter (DAT) levels promoting DA release into the synaptic cleft [150]. DGKη also appears to be downregulated during early myogenic differentiation, and its knockdown inhibits myoblast proliferation without affecting differentiation [151].

DGKκ is linked to fragile X syndrome, and nucleotide variants are associated with hypospadias [152,153]. It is expressed in CD4+ T cells, regulated by the NF-κB pathway, and transferred to target cells, including alveolar epithelial cells, via extracellular vesicles. Increased DGKκ expression in serum extracellular vesicles from patients with sepsis-induced lung injury correlates with sepsis severity and progression [154].

DGKε is unique among diacylglycerol kinases (DGKs) for its specific affinity for DAG with an arachidonic acid (C20:4) at the sn-2 position and either C16:0 or C18:0 at the sn-1 position [50,53,155]. This specificity makes 18:0/20:4 DAG (SAG) a key component of PI and its phosphorylated derivatives, essential for maintaining the PI cycle. Interestingly, SAG binds to a lipoxygenase-like motif in DGKε, raising questions about the roles of its C1 domains, which share features with DAG-binding domains in protein kinase C [50,53]. DGKε activity can exacerbate seizure susceptibility and contribute to Huntington’s disease through altered PI/PPIn levels, while its deletion may lead to obesity in mice [54,89,156,157]. Additionally, DGKE mutations are associated with atypical hemolytic uremic syndrome (aHUS) and membranoproliferative glomerulonephritis, conditions marked by kidney damage due to microvascular thrombosis [113,114,158]. Although the connection between DGKE mutations and aHUS remains unclear, it may involve impaired prostaglandin E2 synthesis, impacting Akt kinase activation in endothelial cells [120,124]. Recent studies show that DGKε regulates Klotho expression partially through the transcription factor Kruppel-like factor (KLF) 15/Klotho pathway [159]. Mice overexpressing DGKE (Rosa26-Dgke⁺/⁺) displayed significant protection against renal ischemia/reperfusion injury (IRI), including reduced tubular cell death, inflammation, and improved kidney morphology [159]. In vitro studies with human renal proximal tubule (HK-2) cells confirmed that DGKE overexpression protected cell death induced by oxygen-glucose deprivation/reoxygenation [159].

DGKζ deficiency impacts type 2 innate lymphoid cell (ILC2)-mediated allergic airway disease. Inhibition of DGKζ confers protection against T cell-mediated allergic airway inflammation by preventing Th2 cell differentiation [160]. DGKζ-deficient mice exhibit reduced ILC2 function and diminished papain-induced airway inflammation compared to wild-type mice [161]. Depletion of DGKζ enhances DAG-regulated transcriptional programs, leading to increased interleukin-2 production and partially mitigating the inhibitory effects of PD-1. Loss of DGKζ results in reduced PD-1 expression and promotes the expansion of cytotoxic CD8+ T cells, even in immunosuppressive environments, suggesting that targeting DGKζ could enhance anti-tumor immunity [162]. In humans, DGKζ promotes breast cancer progression by promoting epithelial–mesenchymal transition (EMT) via the TGFβ signaling pathway [163].

Bioinformatics analysis has shown that DGKι is overexpressed in gastric tumors, correlating with poor prognosis. This overexpression is associated with higher tumor grades, advanced stages, and T classifications [164]. In Dgki-knockout mice, both male and female, sensitivity to histamine was specifically enhanced in vivo, while responses to other pruritogens remained unchanged. This suggests that Dgki selectively regulates sensory neuron and behavioral responses to histamine, without affecting responses to other pruritogenic or algogenic stimuli [165]. In Dgkh and Dgki double-knockout female mice, impaired maternal care was observed, alongside increased manifestations of mania and anxiety. These findings indicate that the combined deletion of Dgkh and Dgki disrupts maternal behavior, suggesting an additive or synergistic effect on behavior when both genes are deleted [166].

DGK-θ is involved in modulating compensatory endocytosis in mouse central nervous system neurons [167] and is influenced by membrane lipids [168,169]. Additionally, the neuronal protein synaptotagmin-1 (Syt1) also regulates DGK-θ [170], highlighting its complex role in neuronal signaling and membrane dynamics. Genome-wide association studies (GWAS) have identified the rs11248060 variant in the DGKQ gene, which increases the risk of Parkinson’s disease (PD) in Caucasian and Han Chinese cohorts [171].

## 6. Inhibitors of DGKs

**Figure 4:**
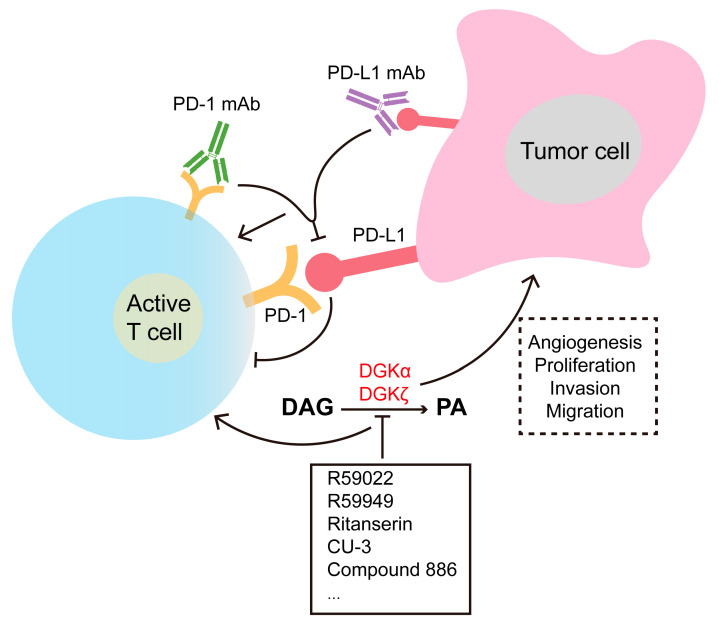
PD-1/PD-L1 blockade and DGK inhibition in cancer treatment. Antibody-based therapies targeting the PD-1/PD-L1 axis have revolutionized cancer treatment, but acquired resistance often develops, typically linked to the upregulation of other inhibitory molecules. The direct effect of DGKα inhibition, which induces apoptosis in cancer cells, coupled with the indirect effect of enhancing cancer immunity through T cell activation, could synergistically damage cancer cells. Additionally, inhibiting DGKζ could further enhance the therapeutic effects of DGKα inhibition. The observed cooperative effect of combining PD-1/PD-L1 blockade with DGKα inhibition provides a promising strategy to improve the efficacy of cancer immunotherapy.

Due to the crucial roles of DGK in the immune system, cell signaling, and lipid metabolism, significant efforts have been made to identify small molecules or other tools that interfere with its activity. R59022 and R59949 are two DGK chemical inhibitors that have been well-characterized and widely used in vitro studies [172,173]. Their structures are similar and they selectively inhibit DGKα activity [173]. Recent studies have identified another chemical with similar properties, ritanserin, a drug used in several clinical trial with no reported toxicity [174]. AMB639752 and ritanserin have also been discovered as new inhibitors through virtual screening (2D/3D) using R59022 and R59949 as templates to explore the chemical space [175,176]. A novel class of promiscuous DGKα/DGKζ inhibitors with submicromolar activity, capable of reducing INFγ production in vitro, have recently been reported [21,177]. Compound 886 preferentially targets DGKζ [176] and CU-3 decreases DGKα activity and PA levels while enhancing TCR-induced production of IL-2 in Jurkat lymphoma cells [178,179,180]. The DGKζ-selective inhibitor ASP1570 similarly enhances NK cell function through DAG-mediated signaling in immunoreceptor-stimulated NK cells, resulting in increased IFNγ production and degranulation in vitro, as well as improved NK cell-mediated tumor clearance in vivo [181]. Recent reports suggest that inhibiting DGKα could help reverse T lymphocyte exhaustion, thereby promoting more effective anti-tumor T cell responses. The observed synergistic effect of combining PD-1/PD-L1 blockade with DGKα inhibition presents a promising strategy to enhance the efficacy of cancer immunotherapy [100]. These findings provide valuable insights for future therapeutic strategies targeting DGK activity (Figure 4).

## 7. Future Prospectives and Conclusions

Although significant research has advanced our understanding of DGKs, their physiological roles in signal transduction and lipid metabolism still require further investigation. DGKs convert DAG to PA, thereby negatively regulating DAG signaling and its associated pathophysiological functions. Through a series of biochemical reactions, PA is recycled to generate new signaling molecules. DGKs act as central switches in terminating DAG signaling and in the resynthesis of membrane phospholipid precursors, thus participating in the regulation of numerous physiological activities. DGKα, as an immune checkpoint, has become well established, and its inhibition has been proposed as a strategy to improve CAR-T cell performance [182]. The classical DAG/PKC pathway is a major signal transduction pathway involving lipid-protein interactions. Recently, new non-lipidic small molecule inhibitors were developed to block the cellular activity of kinases with Src homology 2 (SH2) domains through direct lipid-SH2 domain interactions [183]. Inhibiting the cellular activity of their associated proteins provides new ideas for the development of new inhibitors for DGK-related diseases.

DGKs also play a critical role in lipid metabolism. Specifically, they regulate lipid droplet synthesis by converting 1,2-DAG back into PA. While lipids are primarily synthesized in the ER, DGKε is uniquely localized in the ER [184]. DGKε catalyzes the conversion of 1,2-DAG to PA with a specific affinity for 1-stearoyl-2-arachidonoyl-sn-glycerol, a key backbone of PI. DGKε-KO mice exhibit obesity, insulin resistance and beige adipogenesis when fed a high-fat diet [157]. PI is also an essential precursor for GPI biosynthesis, a crucial post-translational modification of proteins. Further studies are needed to fully elucidate the roles and functions of various DGKs in lipid synthesis. Additionally, due to the close relationship between DGKs, immune cells, and cancer development, identifying new drug targets and small molecule inhibitors remains a significant challenge. This review summarizes the types, cellular localization, functions, and inhibitors of DGKs, providing a comprehensive foundation for future research.

## Figures and Tables

**Table 1:** Main subcellular localization of human DGK isoforms.

	Gene	Substrate Specificity	Main Subcellular Localization
DGKα	*DGKA*	Non-specific	Cytoplasmic
DGKβ	*DGKB*	Non-specific	Postsynaptic membrane, Cytoplasmic
DGKγ	*DGKG*	Non-specific	Golgi apparatus, Cytoplasm
DGKδ	*DGKD*	Non-specific	Plasma membrane
DGKη	*DGKH*	Non-specific	Plasma membrane
DGKκ	*DGKK*	Non-specific	Plasma membrane
DGKε	*DGKE*	sn-2-arachidonoyl (20:4)-DG	Endoplasmic reticulum
DGKζ	*DGKZ*	Non-specific	Nucleus and Plasma membrane
DGKι	*DGKI*	Non-specific	Cytoplasm and nucleus
DGKθ	*DGKQ*	Non-specific	Plasma membrane and nucleus

**Table 2:** The primary DGK-related diseases and symptoms.

	Related Disease	Symptoms	Related References
DGKα	T cell dysfunction; cancer.	T cell hypofunctionality;Tumor growth, invasion and drug resistance; Fibrosis.	[100,104,105,106]
DGKβ	Mood disorder.	Cognitive impairment; mania-like behavior.	[107]
DGKγ	Colon cancer.	Migration and invasion.	[108]
DGKδ	Diabetes; obesity.	Seizures, capillary abnormality, developmental delay, infantile hypotonia and obesity.	[109]
DGKη	Bipolar disorder.	Bipolar disorder.	[110]
DGKκ	Hypospadias.	Intellectual disability; autism.	[111,112]
DGKε	aHUS.	Thrombotic microangiopathy, hemoglobin, microangiopathy.	[113,114,115,116,117,118,119,120,121,122,123,124]
DGKζ	Colon cancer and glioma.	Tumor growth and invasion.	[125,126,127]
DGKι			
DGKθ			

## Abbreviations

GPL: Glycerophospholipids; ER, Endoplasmic Reticulum; G3P, Glycerol-3-phosphate; PA, Phosphatidic acid; DAG, Diacylglycerol; 1,2-DAG, DAG-containing acyl chains at sn-1 and sn-2 positions; PI, Phosphatidylinositol; PS, Phosphatidylserine; PC, Phosphatidylcholine; PE, Phosphatidylethanolamine; TG, Triacylglycerol; LD, Lipid droplet; FA, Fatty acid; CDP-DAG, Cytidine-diphosphate-diacylglycerol; PI4P, Phosphatidylinositol 4-phosphate; PI(4,5)P2, Phosphatidylinositol 4,5-bisphosphate; PI4K, PI 4-kinase; PIP5K, PI4P 5-kinase; PLC, Phospholipase C; DGK, Diacylglycerol kinase; PLD, Phospholipase D; PIS, Phosphatidylinositol synthase; CDS, CDP-DG synthase; PSS, Phosphatidylserine synthase; CEPT, Choline/ethanolamine phosphotransferase; CPT, CDP-choline:1,2-diacylglycerolphosphocholine transferase; DGAT, Diacylglycerol acyl transferase; AGPATs, 1-Acylglycerol-3-Phosphate O-Acyltransferase; GPAT, Glycerol-3-Phosphate Acyltransferase; ACACA, Acetyl-CoA carboxylase 1; ACACB, Acetyl-CoA carboxylase 2; ACSS2, Acyl Co-A synthetase-2; FASN, Fatty acid synthase; HMGCR, 3-hydroxy-3-methylglutaryl-CoA reductase; FDPS, Farnesyl-diphosphate farnesyltransferase 1, SQLE, Squalene monooxygenase; FDFT1, Farnesyl-diphosphate farnesyltransferase 1; PtdIns, Phosphatidyl inositol; PKC, protein kinase C; Gly/Pro, Glycine/Proline; PH, Pleckstrin homology; RVH, Recoverin homology domain; MARCKS, Myristoylated alanine rich protein kinase C substrate phosphorylation site; SAM, Sterile alpha motif; TCR, T-cell receptor; PIP2, Phosphatidylinositol 4,5-bisphosphate; GPI, Glycosylphosphatidylinositol; LPAAT, Lysophosphatidic acid acyltransferase; DGAT, DAG acyltransferase; ATGL, Adipose triglyceride lipase; PD-1/PD-L1, Programmed cell death-1/ligand-1; AP-1, Activator protein-1; ERK, Extracellular signal-regulated kinase; BMDMs, Bone marrow-derived macrophages; mTORC1, mammalian target of rapamycin complex 1; LTD, Long-term depression; PKCγ, Protein kinase C γ; HCC, Hepatocellular carcinoma; SERT, Serotonin transporter; BPD, Bipolar disorder; EGF, Epidermal growth factor; ASK3, Apoptosis signal-regulating kinase 3; DA, Dopamine; Ahus, atypical hemolytic uremic syndrome; AKI, Acute kidney injury; IRI, Ischemia/reperfusion injury; KLF, Kruppel-like factor; ILC2, Influences type 2 innate lymphoid cell; EMT, Epithelial–mesenchymal transition; Syt1, Synaptotagmin-1; GWAS, Genome-wide association studies; PD, Parkinson’s disease.
